# Sphingomyelinase D Activity in *Sicarius tropicus* Venom: Toxic Potential and Clues to the Evolution of SMases D in the Sicariidae Family

**DOI:** 10.3390/toxins13040256

**Published:** 2021-04-01

**Authors:** Priscila Hess Lopes, Caroline Sayuri Fukushima, Rosana Shoji, Rogério Bertani, Denise V. Tambourgi

**Affiliations:** 1 Immunochemistry Laboratory, Butantan Institute, São Paulo 05503-900, Brazil; priscilahess.lopes@gmail.com (P.H.L.); rosana.shoji@butantan.gov.br (R.S.); 2 Special Laboratory of Ecology and Evolution, Butantan Institute, São Paulo 05503-900, Brazil; carolinesayuri@gmail.com (C.S.F.); rogerio.bertani@butantan.gov.br (R.B.); 3 Finnish Museum of Natural History, University of Helsinki, 00014 Helsinki, Finland

**Keywords:** *Sicarius*, *Loxosceles*, spiders, sphingomyelinase D, hemolysis, venoms variations

## Abstract

The spider family Sicariidae includes three genera, *Hexophthalma*, *Sicarius* and *Loxosceles*. The three genera share a common characteristic in their venoms: the presence of Sphingomyelinases D (SMase D). SMases D are considered the toxins that cause the main pathological effects of the *Loxosceles* venom, that is, those responsible for the development of loxoscelism. Some studies have shown that *Sicarius* spiders have less or undetectable SMase D activity in their venoms, when compared to *Hexophthalma*. In contrast, our group has shown that *Sicarius ornatus*, a Brazilian species, has active SMase D and toxic potential to envenomation. However, few species of *Sicarius* have been characterized for their toxic potential. In order to contribute to a better understanding about the toxicity of *Sicarius* venoms, the aim of this study was to characterize the toxic properties of male and female venoms from *Sicarius tropicus* and compare them with that from *Loxosceles laeta*, one of the most toxic *Loxosceles* venoms. We show here that *S. tropicus* venom presents active SMases D. However, regarding hemolysis development, it seems that these toxins in this species present different molecular mechanisms of action than that described for *Loxosceles* venoms, whereas it is similar to those present in bacteria containing SMase D. Besides, our results also suggest that, in addition to the interspecific differences, intraspecific variations in the venoms’ composition may play a role in the toxic potential of venoms from *Sicarius* species.

## 1. Introduction

The Sicariidae Keyserling, 1880 family comprises three genera: *Sicarius* Walckenaer, 1847, *Loxosceles* Heineken and Lowe, 1832 and *Hexophthalma* Karsch, 1879, in which 169 species are included. The large majority of them is in the genus *Loxosceles* (140) that is widely distributed in the New World, Africa and parts of Europe and Asia [1] being considered one of the most relevant spider genus of medical importance. *Sicarius*, with 21 species, is distributed from El Salvador to Chile and Argentina [1]; *Hexophthalma*, with eight species, is found only in Africa [1].

Until recently, the species now included in *Hexophthalma* were considered to belong in *Sicarius* [2]. Therefore, all references to *Hexophthalma* in this text corresponds to previous studies on African *Sicarius*. These three genera comprise specimens notably non-aggressive to humans. *Loxosceles* are small, nocturnal and sedentary spiders [3,4], while *Sicarius* and *Hexophthalma* are larger and spend virtually a lifetime without moving, buried in the sand, between rocks or caves [5].

Despite the similarity of behavior between spiders of these genera, accidents in humans with *Loxosceles* spiders have been increasing in number and area of occurrence [6], while there is only one report of a human accident with *Sicarius* published in Brazil [7]. On the other hand, several studies on *Hexophthalma* show the potential toxicity of these venoms to humans [5,8,9,10,11,12]. Regarding casuistry, habitat is undoubtedly the most important point to be considered, since spiders of the *Loxosceles* genus are synanthropic and live near and inside homes, hidden in furniture, clothes and shoes [3,4], while *Sicarius* and *Hexophthalma* spiders spend a lot of time buried in the ground [5].

*Loxosceles* bites can be considered from mild to severe with systemic manifestations, such as intravascular hemolysis, disseminated intravascular coagulation (DIC) and renal failure [6], that can lead to death. In accidents with *Hexophthalma* spiders, dermonecrotic lesions similar to those seen in loxoscelic accidents have been described [5]. Moreover, studies in animal models have shown the toxic potential of *Hexophthalma* venoms, with the development of skin necrosis, damages in the liver and lungs and biochemical evidence of DIC [5,12].

Although *Hexophthalma* and *Sicarius* venoms are less investigated than the *Loxosceles* ones, it is known that they share an important characteristic: the presence of sphingomyelinases D (SMases D) [13,14,15]. SMases D have already been described as the main players in the development of local lesions and systemic manifestations observed in loxoscelism [16,17,18,19,20,21]. With the change in the African *Sicarius* species to the *Hexophtalma* genus, the presence of SMase D became a characteristic of the Sicariidae Family.

In addition to spider venoms, SMases are also found in other organisms, such as bacteria, ticks and fungi [22,23,24]. Database research has shown that the SMases of the bacteria *Corynebacterium pseudotuberculosis* and *Arcanobacterium haemolyticum* show between 24–30% similarity with the first 30 amino acids of *Loxosceles* toxins [19], as well as molecular weight, isoelectric point and substrate specificity [25]. The bacterial and spider SMases D are functionally similar and considered to have originated from a common ancestor, the glycerophosphodiester phosphodiesterases (GDPD; E.C. 3.1.4.46) [26]. Although there are different or complementary hypotheses about the evolutionary history of SMases D enzymes [2,27,28,29], few studies (or none at all) take into account the similarity or differences in the induction mechanism of effects provoked by SMases D.

**Figure 1:**
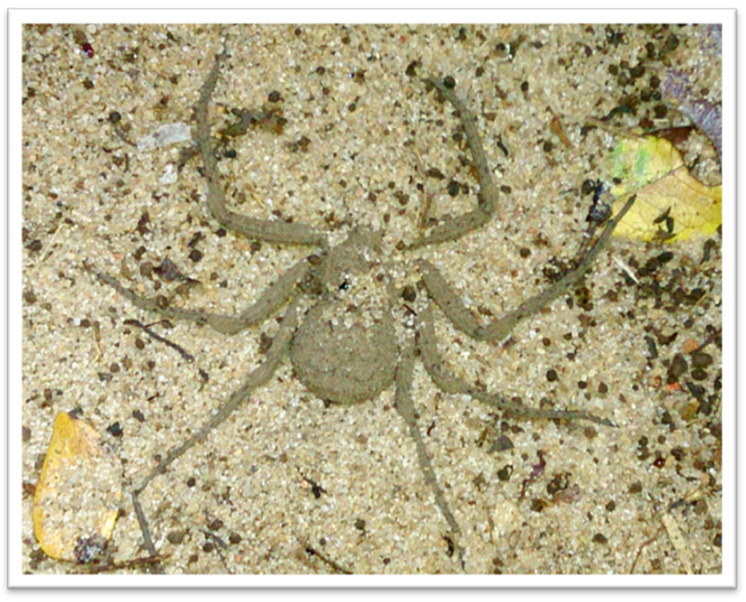
Representative specimen of *Sicarius tropicus*. Photo: Caroline Sayuri Fukushima.

Therefore, the characterization of *Sicarius* species venom can contribute to a better understanding of the evolution of these toxins, as well as the variability of venoms within Sicariidae family and their toxic potential to humans. In the present study, we have analyzed the venom of male and female *Sicarius tropicus* (Mello-Leitão, 1936)*,* regarding the presence and activity of SMases D, and compared them with *Loxosceles laeta* (Nicolet, 1849) venom. Results showed that *S. tropicus* (Figure 1) venom has active SMases D capable of causing cell death and hemolysis dependent on complement system, which, in its induction mechanism, is similar to the mechanism observed in SMases D present in bacteria.

## 2. Results

### 2.1. Sicarius tropicus Venom Biochemical Characterization

**Figure 2:**
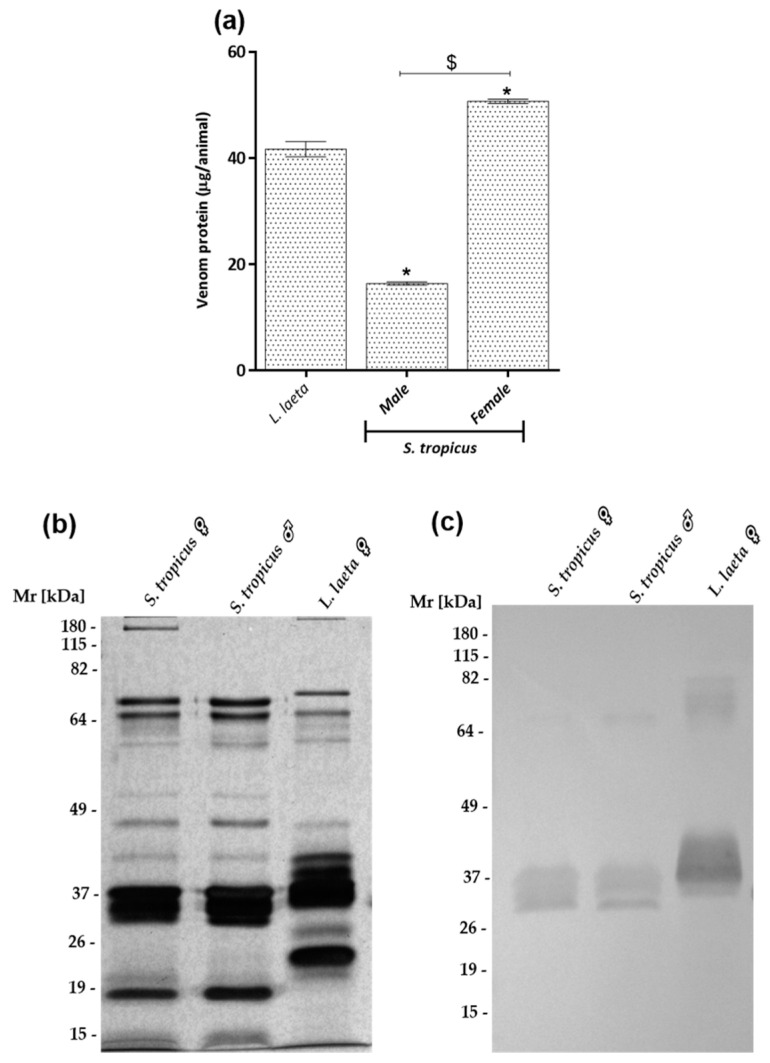
Protein content and immunochemical characterization of *Sicarius tropicus* venom. The protein content of female (*n* = 18) and male (*n* = 25) adult *S. tropicus* spiders venoms samples were determined using the BCA colorimetric method (**a**). Samples of the venoms (10 µg of protein) were subjected to electrophoresis on a 12% SDS-PAGE gel under non-reducing conditions, stained with silver (**b**) or Western blotted (**c**). Blots were revealed with polyclonal serum against SMases D from *L. intermedia* and *L. laeta* diluted 1:2000, followed by anti-horse IgG/AP conjugate (1:7.500) and the reaction developed using NBT/BCIP. Results are expressed as mean ± SE. (*) Significant difference (*p* < 0.05) from *L. laeta* venom; ($) intraspecific variation significantly different (*p* < 0.05).

Analysis of protein content showed intraspecific variability of venoms from *S. tropicus* spiders. Venoms from females of *S. tropicus* (*n* = 18) presented higher protein content (67% higher) than males (*n* = 25), and about 22% higher concentration when compared to female venoms (*n* = 68) from *L. laeta* (Figure 2a).

Electrophoretic analysis of *S. tropicus* venoms revealed a similar profile between male and female venoms (Figure 2b). In comparison to the *L. laeta* venom, a number of slightly different bands (Figure 2b) was observed. Western blot analysis, using a polyclonal antiserum raised against SMases D from *Loxosceles* venoms, revealed the presence of this toxin in *S. tropicus* male and female venoms, as well as in the positive control, the venom of *L. laeta*. Nevertheless, the molecular weight of the bands corresponding to the SMases D vary in these two species (Figure 2c).

**Figure 3:**
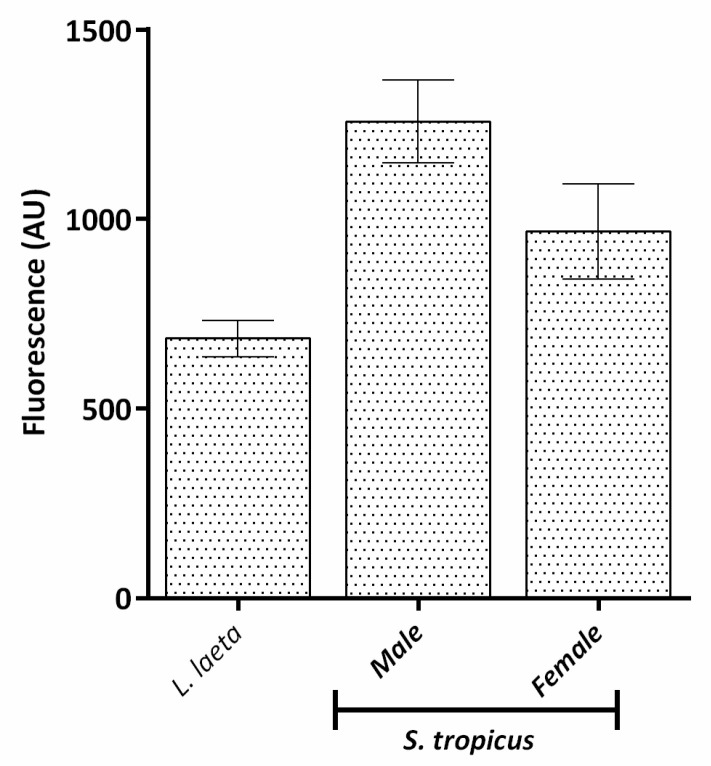
Sphingomyelinase activity of *S. tropicus* venom. Sphingomyelin (10 μM) was incubated with buffer or with *S. tropicus* or *L. laeta*. venoms (10 µg of protein). After 10 min at 37 °C, the formed choline was oxidized to betaine and determined fluorometrically. Results are representative of three independent experiments and expressed as mean ± SE of duplicates.

The analysis of the SMase D activity of *S. tropicus* and *L. laeta* venoms was assessed using sphingomyelin substrate. Results revealed that *S. tropicus* venoms as well as *L. laeta* present active SMases D. However, there was no significant difference between male and female *S. tropicus* venoms. These venoms presented a tendency for greater SMase D activity than *L. laeta* venom (Figure 3).

### 2.2. Female S. tropicus and L. laeta Venoms Reduce the Viability of Human Keratinocytes

**Figure 4:**
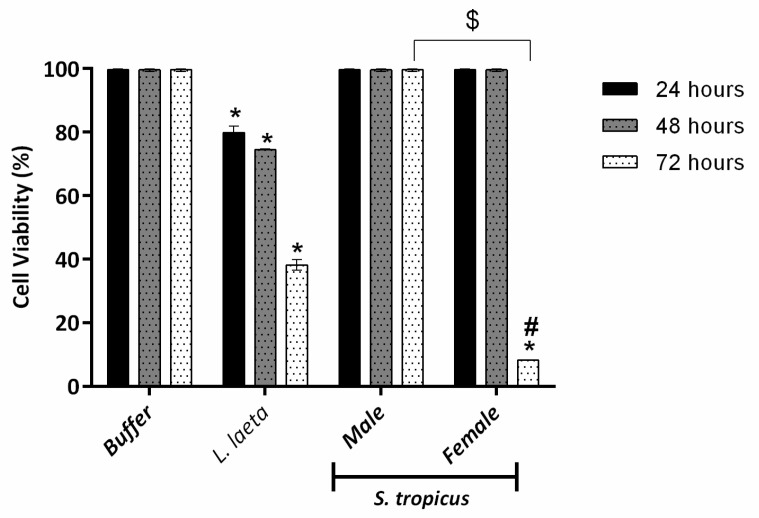
Human keratinocytes viability after treatment with *L. laeta* or *S. tropicus* venoms. HaCaT cells (5 × 10^4^ cells/well) were cultured in 96-well plates with DMEM without FBS for 24 h followed by incubation with venoms (10 μg of protein). After 24, 48 and 72 h, the viability was tested by the MTT method and the readings taken at wavelength of 540 nm. Data are expressed as mean ± SE of duplicates. (*) Significant differences (*p* < 0.05) from negative control (buffer); (#) significant differences from *L. laeta* venom (*p* < 0.05); ($) intraspecific variation significantly different at 72 h period (*p* < 0.05).

We have previously described that *Loxosceles* SMases D reduce the viability of human keratinocytes, contributing to the dermonecrosis development in cutaneous loxoscelism [30]. Analyzing the cytotoxicity of venoms at a concentration of 10 µg of protein, it was possible to observe that only *L. laeta* and female *S. tropicus* venoms were able to reduce cell viability (Figure 4). It is interesting to note that the toxicity profile differs significantly between these two venoms, given *L. laeta* venom gradually reduces cell viability over the 72 h, while the toxic potential of the female *S. tropicus* venom seems to affect cells only after 48 h of treatment. In addition, it was also possible to observe a gender variability of the *S. tropicus* venom, once the male venom did not affect cell viability in any treatment period.

### 2.3. Sicarius tropicus Venom Induces Complement-Dependent Hemolysis in Human Erythrocytes by Different Mechanisms Than L. laeta Venom

**Figure 5:**
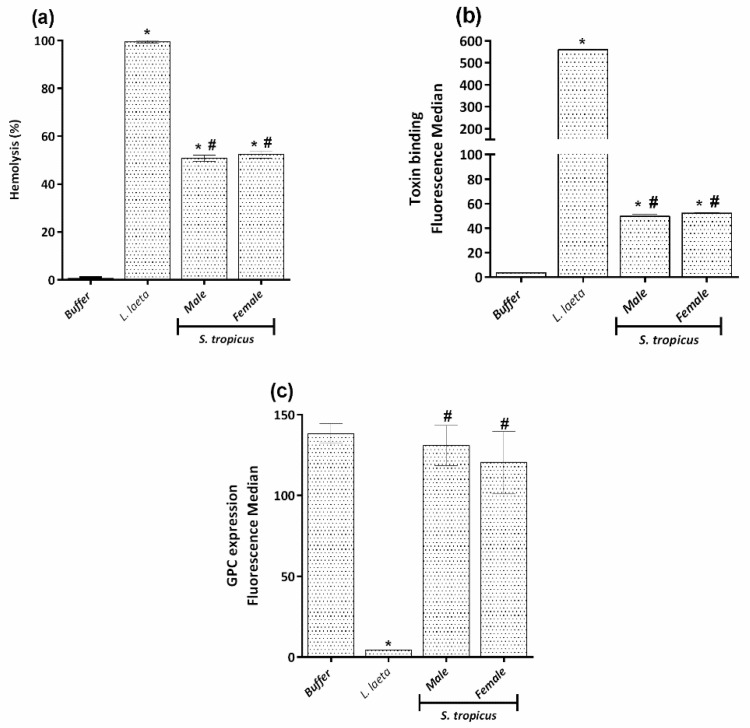
Hemolysis dependent on complement system and its induction mechanism. Human erythrocytes, pre-treated with VBS^2+^, *S. tropicus* or *L. laeta* venoms (5 µg of protein), were incubated with autologous normal human serum. After incubation for 1 h at 37 °C, unlysed cells were spun down; the absorbance of the supernatants was measured at 414 nm and expressed as a percentage of lysis (**a**). The ability of the toxins to insert into the erythrocyte surface was evaluated using a monospecific polyclonal rabbit serum against *Loxosceles* SMase D and analyzed by flow cytometry and expressed as fluorescence median (**b**). Expression of GPC at cell surface analyzed by flow cytometry and expressed as fluorescence median (**c**). Results are representative of three different experiments and expressed as mean ± SE of duplicates. (*) Significant differences (*p* < 0.05) from negative control (buffer); (#) significant differences from *L. laeta* venom (*p* < 0.05).

Our group has demonstrated that SMase D promotes hemolysis by rendering erythrocyte activators of the complement system. SMase D activity on erythrocyte membranes induces the activation of metalloproteases from the Adamlysin family that act by cleaving the sialic acid-rich extracellular domains of glycophorins A, B and C (GPA, GPB and GPC) from erythrocytes, thereby rendering these cell activators of the alternative pathway [19,20]. Comparing the male and female *S. tropicus* venoms with *L. laeta* venom, it was possible to observe that *S. tropicus* venoms induce about 50% of hemolysis, dependent on the autologous complement activation (Figure 5a), while *L. laeta,* at the same concentration, reaches 100% of hemolysis.

Flow cytometric analysis of erythrocytes treated with *S. tropicus* venoms revealed the presence of SMase D bound to the cell membrane (Figure 5b), but significantly in lower proportion than on the cells treated with the *L. laeta* venom. However, when analyzing the GPC expression on the erythrocyte’s membranes, it was observed that the cells do not differ from the negative control treated only with buffer, i.e., there was no removal of the extracellular portion of the GPC (Figure 5c).

**Figure 6:**
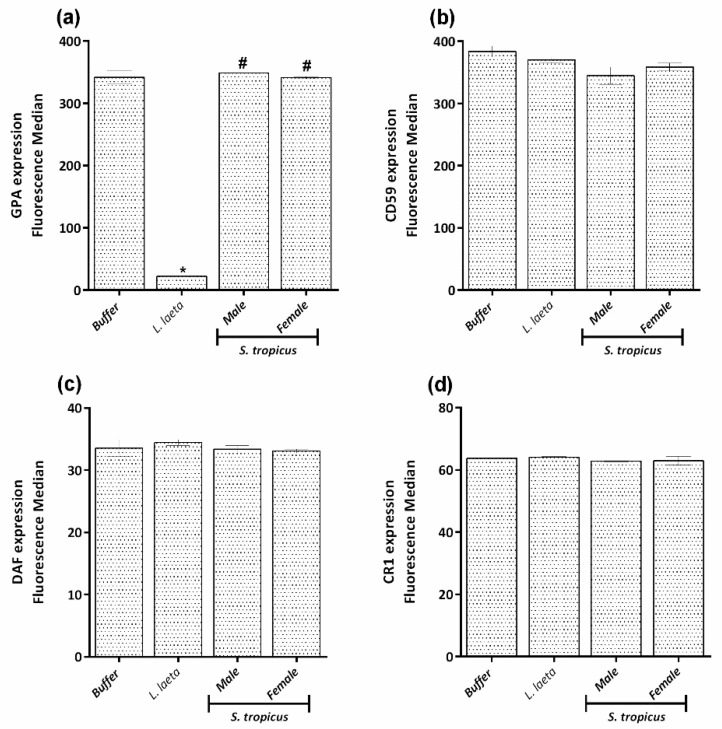
The expression of GPA and complement system regulators (CD59, DAF and CR1) in cells treated with *S. tropicus* venom. Human erythrocytes, pre-treated with VBS^2+^, *S. tropicus* or *L. laeta* venoms (5 µg of protein) and the expression of GPA (**a**), CD59 (**b**), DAF (**c**) and CR1 (**d**), were analyzed by flow cytometry and expressed as fluorescence median. Results are representative of three different experiments and expressed as mean ± SE of duplicates. (#) significant differences from *L. laeta* venom (*p* < 0.05)

To confirm this result, we also analyzed the GPA expression. Figure 6a shows that the cells treated with *S. tropicus* venoms also do not have reduced GPA expression, unlike what was observed for *L. laeta*. Together, these results indicate that the hemolysis induced by the *S. tropicus* venom occurs by a different mechanism than that observed for *Loxosceles* venoms.

In order to elucidate the mechanism of hemolysis, we investigated the expression of complement system regulators (DAF, CD59, CR1) in the cells treated with *S. tropicus* venoms as well as those treated with *L. laeta* venom. Figure 6b–d shows that cells treated with *S. tropicus* or *L. laeta* venoms have no reduction in the expression of those regulators.

### 2.4. PLA_2_ and PLC Activities Are Not Involved in the Hemolysis Induced by the Venom of S. tropicus

**Figure 7:**
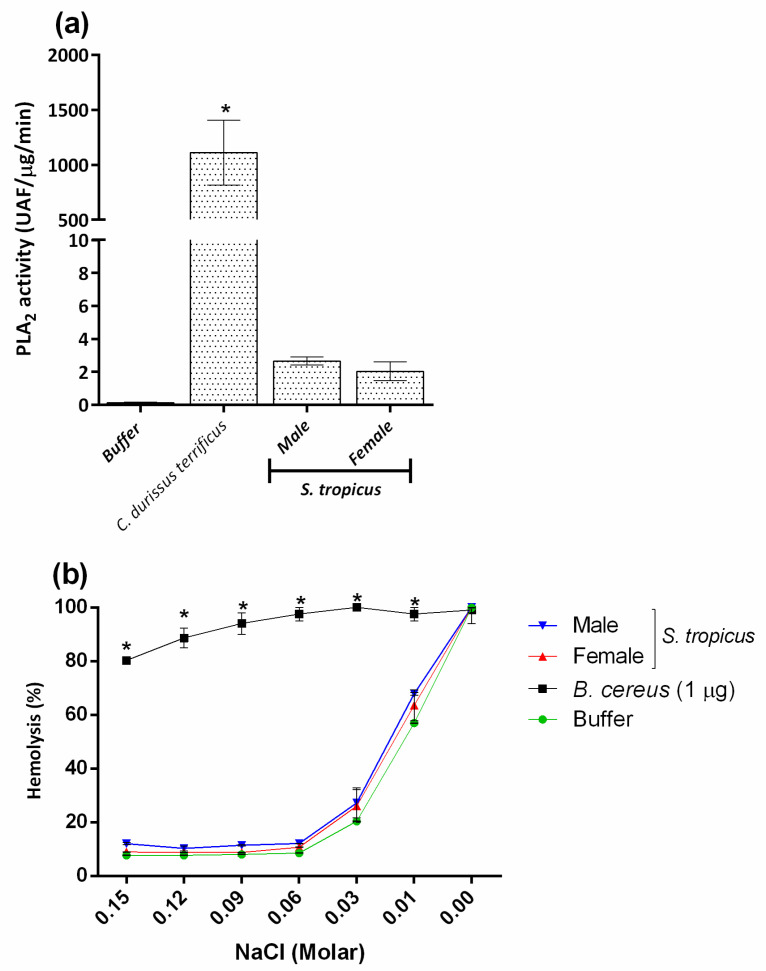
PLA_2_ and PLC activities of *S. tropicus* venom. Samples of *S. tropicus* venom (1 μg of protein) were incubated at 37 °C with a phospholipid mix that containing 10 mM of phosphatidylcholine and 10 mM of phosphatidylglycerol. Increased fluorescence was measured for 10 min. As a positive control, *C. d. terrificus* snake venom (0.5 μg of protein) was used. The results are expressed as specific activity (UF per μg of venom per minute) ± SE (**a**). Human erythrocytes, pre-treated with VBS^2+^, *S. tropicus* (5 µg of protein) or *B. cereus* PLC (1 µg of protein), were incubated for 1h at 37 °C with saline solution with decreasing concentrations of NaCl (0.15 to 0.0 M NaCl). After the incubation, the plates were centrifuged and the supernatant was analyzed at λ414nm, and the results were expressed as a percentage of lysis (**b**). Results are representative of three different experiments and expressed as mean ± SE of duplicates. (*) Significant differences (*p* < 0.05) from negative control.

The hemolytic capacity of PLA_2_ has been previously described [31,32]. Thus, in order to verify whether the hemolysis induced by *S. tropicus* venoms was due to a possible PLA_2_ activity, we have evaluated the presence of this activity in the venom using the venom of the *Crotalus durissus terrificus* (Laurenti, 1768) snake as positive control. Figure 7a shows the high PLA_2_ activity presented by the *C. d. terrificus* venom and the absence of this activity in *S. tropicus* venom.

Another enzyme whose activity could transform erythrocytes susceptible to osmotic lysis is the PLC, as previously demonstrated for SMases C from *Bacillus cereus* [33,34], which was used here as positive control. Figure 7b shows that the male and female venoms from *S. tropicus*, differently from SMases C from *B. cereus,* were not able to make erythrocytes susceptible to osmotic lysis.

### 2.5. Sicarius tropicus Venom Induces Phosphatidylserine Exposition in the Erythrocytes Outer Membrane Leading to Complement-Dependent Lysis

**Figure 8:**
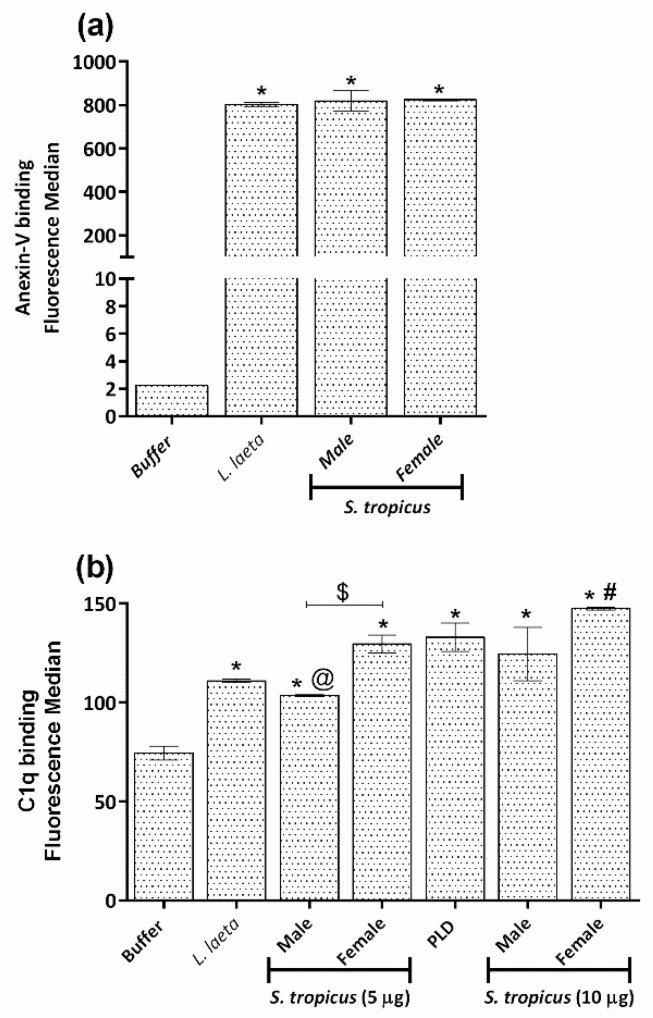
Phosphatidylserine exposure and C1q binding to the surface of erythrocytes treated with *S. tropicus* venom. Human erythrocytes, pre-treated with VBS^2+^, *S. tropicus* (5 and 10 µg of protein) or *L. laeta* (5 µg of protein) venoms and PLD from *C. pseudotuberculosis* (5 µg of protein). The exposure of phosphatidylserine was analyzed by flow cytometry using Annexin-V-FITC. The results are expressed as fluorescence median (**a**). The erythrocytes were incubated with 50 µg/mL of purified C1q, and the binding was analyzed by flow cytometry using anti-human C1q-FITC. The results are expressed as fluorescence median (**b**). Results are representative of three different experiments and expressed as mean ± SE of duplicates. (*) Significant differences (*p* < 0.05) from negative control. (@) significant differences (*p* < 0.05) from positive control (PLD); (#) significant differences from *L. laeta* venom (*p* < 0.05); ($) intraspecific variation significantly different (*p* < 0.05).

Negatively charged lipids, such as phosphatidylserine, are normally only expressed in the inner leaflet of the erythrocyte plasma membrane and are only exposed upon loss of membrane asymmetry [35]. It is known that the exposure of phosphatidylserine in the erythrocytes outer membrane facilitates the binding of the complement C1q component and the activation of the classic complement pathway, as we have already demonstrated for *Loxosceles* venoms and bacterial PLDs [36,37]. Flow cytometry experiments showed that male and female venoms from *S. tropicus* induce phosphatidylserine exposure in the outer membrane layer, as well as the venom from *L. laeta* venom (Figure 8a).

To assess the role of C1 as an initiating factor in *S. tropicus* venoms induced C-susceptibility, venoms-treated erythrocytes were incubated with purified C1q, and binding was measured by flow cytometry. The level of C1q detected on the erythrocytes treated with the spiders’ venoms were significantly different from the negative control and similar to the one induced by the positive control, the PLD from the bacteria *C. hemolyticum*. Moreover, the female venom from *S. tropicus* was able to induce a higher C1q deposition on the human erythrocyte cell membrane than the male venom (Figure 8b).

## 3. Discussion

Spider venoms are complex mixtures. Intra- and interspecific variations have been poorly evaluated in this group of arthropods. Our results showed that male venoms of *S. tropicus* presents lower protein content than *L. laeta*, while the *S. tropicus* female venom has a higher protein content, although still much lower than that demonstrated for another Brazilian species, *Sicarius ornatus* Magalhães, Brescovit and Santos, 2013, in which, both male and female venom have a protein content approximately ten times higher than in *L. laeta* [15]. Binford and Wells (2003) also showed a higher protein content in the venom of the African species *S. hahni* and *S. testaceus* (now both species are considered synonyms as *Hexophthalma hahni* [38]) compared to *L. laeta*. Regarding another South American species, *S. thomisoides* Walckenaer, 1847, a widely distributed Chilean species, also showed, at least in females, a higher protein content in the venom (at least five times higher) [39].

Another interesting point is the gender variation in the protein content of *S. tropicus* venoms. Regarding this, it was possible to observe a three times higher protein amount in female venom than in male venom (Figure 2a). The production of larger protein content by females was correlated with their larger body size and possibly to a higher toxic potential [40]. However, it is unclear whether the amount of protein in the venom gland is important for the toxic potential.

SDS-PAGE analysis did not show gender variation in *S. tropicus* venoms profiles. Besides, only a few differences in terms of number and intensity of bands were observed when comparing *S. tropicus* and *L. laeta* venoms (Figure 2b). What draws the highest attention is the presence of majoritarian bands in the range of 32–35 kDa, corresponding to the molecular weight of SMases D. The identity of these bands was confirmed by the use of a polyclonal serum against the purified SMases D from *L. intermedia* and *L. laeta* venoms, which strongly recognized the bands in the molecular weight range of 32–35 kDa, indicating the presence of this toxin in *S. tropicus* venom (Figure 2c). The same indications of SMases D presence, observed in the electrophoretic and Western blot profiles, were also identified in the South American species *S. ornatus* [15] and *S. thomisoides* [39]. Zobel-Thropp and collaborators (2010) compared venoms from two species of *Hexophthalma* and one species of *Sicarius* from Central America by 2D eletrophoresis and observed distinct patterns for each one, but also observed regions with overlapped and high-density spots for all species, identified as expressed products of the *SicTox* genes, which encode the SMases D [41].

Although there are few reports of human envenomation by *Sicarius* and *Hexophthalma* spiders, studies have shown the presence of active SMases D in the venoms of several species of these genera, mainly from the African *Hexophthalma* species [14]. The authors also showed that species in the American *Sicarius* have SMase D activity at least three times lower or undetectable, compared to those on the *Hexophthalma* from the African continent [14]. In contrast, our group showed that the South American species, *S. ornatus*, has SMase D activity quite similar to the *L. laeta* venom [15], and recently, positive SMase D activity has also been demonstrated in the *S. thomisoides* venom, although comparatively less than that of the *L. laeta* venom [39]. Corroborating these previous studies, the results reported here indicate the presence of active SMases D in *S. tropicus* venom. Although not statistically significant, it seems to be a trend of variation between males and females, as already described for *S. ornatus* venom [15]. The differences between these studies may be related to interspecific variations in the venoms, as well as possible differences in the assembly of the venom pools analyzed in the different studies.

Active SMases D are essential for the development of dermonecrotic lesions, and venoms of *Loxosceles*, *Hexophthalma* and *Sicarius* have already been reported to induce dermonecrosis in animal models and humans [5,7,19,30,39,42,43,44]. Our group has successfully been using human keratinocytes of the HaCaT lineage as a model for in vitro study of the dermonecrotic capacity of venoms [15,30,45,46,47]. Here, we showed that although *L. laeta* venom promotes increased keratinocytes death in a time-dependent way, the *S. tropicus* female venom was able to affect the viability of these cells only after 48 h of treatment. The male venom was not able to induce cell death in any of the tested periods. This same pattern was also observed for *S. ornatus* [15] and *L. intermedia* venoms [40]. Indeed, we have demonstrated that female *L. intermedia* venom has a higher ability of inducing dermonecrotic lesions in rabbits than the male venoms, the result of which was correlated with the higher amount of SMases D in the female venom [40]. However, the lack of cytotoxic effect of male venoms does not correlate positively with the SMase D activity, perhaps because the method used to measure the activity does not fully reflect the enzyme’s in vivo activity.

Regarding systemic effects, there are no reports of *Sicarius* or *Hexophthalma* venoms inducing hemolysis or DIC in humans, although experimentally, interspecific variations have been observed in the *Sicarius*/*Hexophthalma* genus regarding this activity. Our findings showed that *S. tropicus* venoms are capable of inducing a maximum of 50% of hemolysis, even at higher concentrations (data not shown), whereas *S. ornatus* venom, at the same initial concentration of 5 µg was able to induce more than 60% of hemolysis [15], and the venom from females of the species *S. thomisoides* induces a hemolytic pattern quite similar to the venom of *L. laeta* [39]. Such variations have also been demonstrated for species of *Hexophthalma* from the African continent, as shown by Newlands and Atkinson [5], the *S. albospinosus* (now *H. albospinosa* (Purcell, 1908)) venom is capable of inducing systemic effects such as DIC in rabbits. In contrast, the *S. testaceus* (now *H. hahni*) venom did not induce such effect [12]. Curiously, this pattern of maximum of 50% of hemolysis induction was found for SMases D present in the bacteria *C. pseudotuberculosis* [36].

We have also previously described the molecular mechanism of hemolysis induced by *Loxosceles* venoms. We have shown that SMases D bind to the erythrocyte membrane, inducing the activation of endogenous membrane metalloproteinases [19,20]. These proteinases were able to cleave the extracellular portions of glycophorins A, B and C, rich in sialic acid, and once removed, cells become susceptible to lysis of autologous complement by the alternative pathway [20]. Curiously, when evaluating the mechanism of hemolysis induced by *S. tropicus* venoms, it was possible to observe that the binding of the toxin to the erythrocyte membrane, even though different from the negative control, was 10 times lower than in cells treated with *L. laeta* venom. This difference could be partly attributed to antigen recognition, due to the use of a serum produced against SMase D from *Loxosceles* venom. However, when analyzing the GPC content, it was observed that its expression remained statistically unchanged in relation to the cell treated only with buffer. To confirm this result, we also analyzed the GPA content, since this is the main sialoglycoprotein in human erythrocytes, and again, unlike the *L. laeta* venom, it was observed that the *S. tropicus* venoms did not induce any change in the expression of GPA. These results, together, demonstrate that the *S. tropicus* venom induces complement-dependent hemolysis, but by a different mechanism from that known for the *Loxosceles* venoms [19,20,36] and for the *S. ornatus* venom [15]. Interestingly, SMases D from bacteria are also not able to induce the removal of glycophorins from erythrocytes surface [36].

*Sicarius*’ venoms have not been extensively studied regarding hemolysis and their induction mechanism. Thus, in order to elucidate this mechanism, we also investigated the expression of complement system regulators in the cell membrane. The results showed that the expression of these regulators remained unchanged in the cells treated with the venoms, as also observed with *Loxosceles* venoms [20] and bacterial PLD [36]. Activities of other enzymes such as PLA_2_ and PLC can also lead to hemolysis, either directly, by disrupting the cell after cleavage of membrane phospholipids [31,32], as in the venom of the spider *Phoneutria boliviensis* (F. O. Pickard-Cambridge, 1897) [48], or by making cells more susceptible to osmotic lysis, as observed for SMases C (PLC) from *Bacillus cereus* [33,34]. However, none of these enzymes appeared to be present or contribute to the hemolysis mechanism of *S. tropicus* venom (Figure 8).

As mentioned before, SMases D are present in other organisms besides the venoms of *Loxosceles*, *Sicarius* and *Hexophtalma* spiders. The PLD present in bacteria as *C. pseudotuberculosis* shares some structural homology and the ability of promoting clinical symptoms similar to the ones produce by *Loxosceles* SMases D [25,49]. We have previously demonstrated that PLD from *C. pseudotuberculosis* induce 50% of complement-dependent hemolysis without altering the expression of GPC, GPA and regulators, a pattern very similar to the results obtained here. Then, based on these similarities, we investigated the ability of *S. tropicus* venom to induce phosphatidylserine (PS) exposure in the outer membrane of erythrocytes, and it was possible to observe that as *L. laeta* venom, male and female venoms from *S. tropicus* were able to induce the exposure of this phospholipid to the outer layer of cells (Figure 8a).

As previously described, the activity of *Loxosceles* SMases D and bacteria PLD in the membrane induces loss of asymmetry and exposure of PS, which functions as an activating factor of the classical complement pathway, demonstrated by the deposition of components of this pathway on the cell surface [36,37]. The evaluation of C1q deposition on the erythrocyte membrane allowed us to observe the binding of this C-component on cells treated with the *S. tropicus* venoms but not on cells treated with buffer. Interestingly, this event was similar to that observed in cells treated with PLD of *C. pseudotuberculosis*, used as a positive control (Figure 8b). It was also possible to observe a gender difference in this action, since cells treated with female venoms presented a higher C1q binding. Thus, our results suggest that SMases D of *S. tropicus* induce hemolysis in a similar way to bacterial PLD, and the greater hemolytic potency of the *Loxosceles* venoms is due to the ability to also activate the complement alternative pathway.

Studies on the evolutionary history of SMases D have been carried out, and the main evidence indicates that SMases D of spiders and bacteria evolved independently of a common ancestor of the family of glycephosphoryl diester phosphodiesterases (GDPDs), which has representatives in the main organisms [27]. The authors also found a structural motif conserved in SMases D of bacteria and spiders that is not present in GDPDs, which implies the evolution of these SMases may be explained by lateral transfer of genes [27]. The discovery of SMase D genes in ticks [23] reinforce the hypothesis of an ancient presence in arachnids, and therefore, the lateral transfer moved from arachnid to bacteria [28]. On the other hand, Dias-Lopes and collaborators [24] described SMases D genes in fungi and performed a new phylogenetic analysis including this taxon. The results showed that SMases D may have evolved by convergence, independently of an ancestral GDPD in arthropods and fungi. The nucleotide sequences of bacterial SMases showed great similarity with those found in fungi. Therefore, the authors suggest that the lateral gene transfer event occurred from fungi to bacteria. Further studies are needed to better understand the evolutionary history of these enzymes. However, their toxic activity has not been taken into account.

Regarding this, Pedroso et al. [29] showed that SMases D evolved by natural selection towards greater toxicity. In the SMases D sequences of *Sicarius*/*Hexophtalma* species analyzed by them, only class II SMases D were found. Class II SMases D are considered less toxic when compared to Class I [50], which were found only in some of the analyzed *Loxosceles* species. The SMases D of bacteria, for not presenting cysteine residues at homologous sites when compared to the other sequences, were classified neither as class I nor II [29]. Although our results indicate similar toxic activity between SMase D of *S. tropicus* and bacterial ones, duplication and loss of genes prevent SMases D from being the best marker for investigating the relationship of species [27].

## 4. Conclusions

In conclusion, our results show that there is toxic potential for human accidents in *Sicarius tropicus* venom, which presents active SMases D presenting some toxic molecular mechanisms similar to bacterial PLD, capable of causing human skin cell death and complement-dependent hemolysis.

## 5. Material and Methods

### 5.1. Reagents, Antibodies and Buffers

Tween-20, bovine serum albumin (BSA), paraformaldehyde, 3-(4,5dimethylthiazol-2yl)-2,5 diphenyltetrazolium bromide (MTT), sphingomyelin from bovine brain (SM), lysophosphatidylcholine, choline oxidase, horseradish peroxidase (HRPO), 3-(4-hydroxy-phenyl) propionic acid and purified PLC from *Bacillus cereus* were purchased from Sigma Co. (St. Louis, MO, USA).BCIP (5-bromo-4-chloro-3-indolyl-phosphate) and NBT (nitroblue tetrazolium) were bought from Promega Corp. (Madison, WI, USA). The complement component C1q was purchased from CompTech (Complement Technology Inc., Tyler, TX, USA). Rabbit anti-mouse IgG-FITC, goat anti-rabbit IgG-FITC and mouse anti-horse IgG-alkaline phosphatase (AP) were purchased from Sigma Co. (St. Louis, MO, USA). Mouse anti-rabbit IgG-Alexa-488 was obtained from Abcam (Cambridge, UK). Mouse monoclonal antibody against GPC (Bric4, extracellular epitope aa 16–23), GPA (Bric256, extracellular part epitope aa 41–58), CD59 (Bric229), DAF (Bric216) were obtained from IBGRL (Bristol, UK). Monospecific rabbit polyclonal sera against CR1 was produced in-house [20]. Horse serum against SMases D from *L. intermedia* and *L. laeta* venom was obtained as previously described [51]. Rabbit serum against SMase D from *L. intermedia* venom was obtained as previously described [18], and recombinant PLD from *Corynebacterium pseudotuberculosis* was obtained as described [36]. Anti-human C1q produced in mouse was obtained from Quidell (San Diego, CA, USA). The buffers were: HEPES-buffered Saline (HBS) (10 mM Hepes, 140 mM NaCl, 5 mM KCl, 1 mM CaCl_2_, 1 mM MgCl_2_ pH 7.4); Phosphate-buffered Saline (PBS) (8.1 mM Na_2_HPO_4_, 1.5 mM KH_2_PO_4_, 137 mM NaCl, 2.7 mM KCl, pH 7.4); Fluorescence Activated Cell Sorter (FACS) buffer (PBS, 1% BSA, 0.01% sodium azide); Veronal-buffered Saline (VBS^2+^) (10 mM NaBarbitone, 0.15 mM CaCl_2_ and 0.5 mM MgCl_2_ pH 7.4); Alsever’s old solution (114 mM citrate, 27 mM glucose, 72 mM NaCl, pH 6.1).

### 5.2. Spiders and Venoms

The spider specimens of *Sicarius tropicus* used here were collected in the locality of Seridó (S 06°34′ W37°15′), in the State of Rio Grande do Norte, Brazil, under the collection authorization SISBio nº 41006-2. These specimens were kept in the Laboratory of Immunochemistry, Instituto Butantan, Brazil, together with the adult females of *L. laeta* (capture and maintenance licenses from IBAMA, Brazil, number 45166-6). *S. tropicus* adult males were identified by having a fully developed palpal copulatory organ and females by the epigastric furrow presenting a clearly visible opening.

The venoms were obtained by electrostimulation by the method of [52], with slight modifications. Briefly, 15–20 V electrical stimuli were repeatedly applied to the spider sternum, and the venom drops were collected with a micropipette in PBS, aliquoted and stored at −20 °C. The protein content of the venoms was evaluated using the BCA Protein Assay Kit (Pierce Biotechnology, Waltham, MA, USA). Authorization of access to genetic resources register n. AEE9AEA (11/01/2018) and AD50761 (11/05/2018) was provided by National System of Management of Genetic Heritage and Associated Traditional Knowledge (SisGen).

The venom of *L. laeta* is very well characterized and has been used as reference for the characterization of venoms from other species of *Loxosceles* or *Sicarius* [15]. Likewise, in this study, the venoms of *S. tropicus* were compared to the venom of *L. laeta*.

### 5.3. Electrophoresis and Western Blotting

The electrophoretic profile of the venoms (10 µg of protein) was determined on 12% SDS-PAGE acrylamide gel [53] in non-reducing conditions. To visualize the protein bands, silver stain was used [54]. Alternatively, to evaluate the presence of SMases D, proteins were transferred to nitrocellulose membranes [55], and the reaction was revealed using a horse serum anti-SMases D from *L. intermedia* and *L. laeta* venoms (diluted 1:2000 in PBS/1% BSA) followed by specific secondary antibody, mouse anti-horse IgG-AP (1:7500 in PBS/1% BSA) for 1 h at room temperature. After, blots were developed using NBT/BCIP according to the manufacturer’s instructions.

### 5.4. Enzymatic Activity

The SMase D activity of the venoms was evaluated by fluorimetry as described [56]. In sum, samples of venoms (10 μg of protein) were incubated with the substrate sphingomyelin from bovine brain for 10 min at 37 °C. After this period, the reaction was developed with a mixture consisting of 1 unit of choline oxidase/mL, 0.06 units of horseradish peroxidase/mL and 50 μM of 3-(4-hydroxy-phenyl) propionic acid in HBS for 10 min at 37 °C. The choline liberated in the reaction is oxidized to betaine and H_2_O_2_, and this product was determined at λem = 405 nm and λex = 320 nm, in a spectrofluorimeter FLUOstar Omega (BMG Labtech, Offenburg, Germany).

### 5.5. Normal Human Serum and Erythrocytes

The human blood samples to obtain the serum or erythrocytes were collected from healthy donors who knew the objectives of the study and signed the corresponding informed consent form approved by the ethics committee (CAAE: 48350315.6.0000.5467). For normal human serum (NHS), blood samples were collected without anticoagulant and were allowed to clot for four hours at 4 °C. After centrifugation, normal human serum was collected and stored at −80 °C. Differently, blood samples to obtain erythrocytes (E) were collected in anticoagulant Alsever’s old solution.

#### 5.5.1. Treatment of Erythrocytes with Venoms

A suspension of human erythrocytes was prepared by washing cells three times in PBS and resuspended it at 2% in VBS^2+^. One milliliter of this suspension was incubated with venoms (5 µg of protein/mL) or VBS^2+^ for 30 min at 37 °C under agitation. After this period, the complement (C)-dependent hemolytic capacity of the venoms was analyzed as described [18]. To analyze the expression of glycophorins and the binding of SMase D, erythrocytes were prepared for flow cytometry as described.

#### 5.5.2. Flow Cytometry

Samples of human erythrocytes suspension (25 μL) treated with venoms, as described above, were incubated for 30 min with mouse monoclonal antibody against GPC (Bric, 1:250), GPA (Bric256, 1:250), CD59 (Bric229, 1:250), DAF (Bric216, 1:100) or with rabbit serum anti-CR1 (1:50) or against SMase D from *L. intermedia* (1:200) diluted in FACS buffer. After, cells were washed and incubated for more 30 min with the secondary antibodies anti-mouse FITC or anti-rabbit Alexa 488 for 30 min. Then, cells were washed, fixed in FACS buffer containing 1% paraformaldehyde and analyzed by flow cytometry (FACScanto, Becton Dickinson, CA, USA). Additionally, the erythrocytes treated with the venoms were incubated for 30 min at 37 °C with purified C1q (50 µg/mL in VBS^+2^ buffer) and then with the antibodies anti-human C1q produced in mouse (1:100 in FACS buffer), followed by anti-mouse IgG-FITC (1: 100 in FACS buffer), and analyzed for C1q binding to the membrane by flow cytometry. Besides, the exposition of phosphatidylserine on the surface of cells treated or not with the venoms was analyzed using Annexin-V-FITC Apoptosis Detection Kit II (BD Pharmigen), according to the manufacturer’s instructions.

#### 5.5.3. Spontaneous Osmotic Lysis

Human erythrocytes treated with *S. tropicus* or *L. laeta* venoms, as described above, were incubated for 1 h at 37 °C with saline solution with decreasing concentrations of NaCl (0.15 to 0.0 M NaCl). After the incubation, the plates were centrifuged, and the supernatant was analyzed at λ414nm in a plate spectrophotometer. Purified PLC from *Bacilus cereus* (1 µg), able to alter the cell membrane and transform red cells susceptible to osmotic lysis, was used as positive control.

### 5.6. Human Keratinocytes Cultures

Human keratinocytes from HaCaT lineage (Cell Bank of Rio de Janeiro, Rio de Janeiro, Brazil) were cultivated in DMEM medium (Gibco-BRL, Gaithersburg, MD, USA), containing 10% (vol/vol) heat-inactivated (56 °C, 30 min) fetal bovine serum (FBS; Cultilab, São Paulo, Brazil) and 1% (*v*/*v*) of antibiotics (100 IU of penicillin/mL, and 100 IU of streptomycin/mL) at 37 °C in humidified air with 5% CO_2_.

#### Viability Assay

In 96-well plates, cells were plated at the density of 5 × 10^4^ cells/well. After 24 h, the medium was changed, and cells were maintained overnight in DMEM without FBS, followed by incubation with venoms (10 μg of protein) for 24, 48 and 72 h. DMEM without FBS was used as negative control. After incubation, the viability of the cells was evaluated by the method of MTT (3-(4,5-dimethylthiazol-2-yl)-2,5-di-phenyltetrazolium bromide) [57].

### 5.7. Phospholipase A_2_ Activity

The EnzChek^TM^ Phospholipase A_2_ Assay Kit was used to analyze a possible PLA_2_ activity in *S. tropicus* venoms (1 µg), following the manufacturer’s recommendations. *Crotalus durissus terrificus* snake venom (0.5 µg) was used as positive control.

### 5.8. Statistical Analysis

Data were expressed as mean ± standard error and statistically analyzed with GraphPad Prism, version 6.1 for Windows (San Diego, CA, USA). The data distribution was checked using the D’Agostino-Pearson omnibus normality test. For comparisons, the following tests were used: one-way ANOVA and multiple comparisons performed by post hoc Tukey HSD; two-way ANOVA followed by post hoc Bonferroni test and Student’s t Test.

## Data Availability

All datasets generated for this study are included in the article.
